# Anetumab ravtansine inhibits tumor growth and shows additive effect in combination with targeted agents and chemotherapy in mesothelin-expressing human ovarian cancer models

**DOI:** 10.18632/oncotarget.26135

**Published:** 2018-09-25

**Authors:** Maria Quanz, Urs B. Hagemann, Sabine Zitzmann-Kolbe, Beatrix Stelte-Ludwig, Sven Golfier, Cem Elbi, Dominik Mumberg, Karl Ziegelbauer, Christoph A. Schatz

**Affiliations:** ^1^ Bayer AG Preclinical Research, Pharmaceuticals, Berlin 13353, Germany; ^2^ Bayer AG Preclinical Research, Pharmaceuticals, Wuppertal 42096, Germany; ^3^ Bayer US LLS, Whippany, NJ 07981, USA

**Keywords:** mesothelin, anetumab ravtansine, antibody-drug conjugate, copanlisib, ovarian cancer

## Abstract

Despite the recent advances in the treatment of ovarian cancer, it remains an area of high unmet medical need. Epithelial ovarian cancer is associated with high levels of mesothelin expression, and therefore, mesothelin is an attractive candidate target for the treatment of this disease. Herein, we investigated the antitumor efficacy of the mesothelin-targeting antibody-drug conjugate (ADC) anetumab ravtansine as a novel treatment option for ovarian cancer in monotherapy and in combination with the antitumor agents pegylated liposomal doxorubicin (PLD), carboplatin, copanlisib and bevacizumab. Anetumab ravtansine showed potent antitumor activity as a monotherapy in ovarian cancer models with high mesothelin expression. No activity was seen in mesothelin-negative models. The combination of anetumab ravtansine with PLD showed additive anti-proliferative activity *in vitro*, which translated into improved therapeutic *in vivo* efficacy in ovarian cancer cell line- and patient-derived xenograft (PDX) models compared to either agents as a monotherapy. The combination of anetumab ravtansine with the PI3Kα/δ inhibitor copanlisib was additive in the OVCAR-3 and OVCAR-8 cell lines *in vitro*, showing increased apoptosis in response to the combination treatment. *In vivo*, the combination of anetumab ravtansine with copanlisib resulted in more potent antitumor activity than either of the treatments alone. Likewise, the combination of anetumab ravtansine with carboplatin or bevacizumab showed improved *in vivo* efficacy in the ST081 and OVCAR-3 models, respectively. All combinations were well-tolerated. Taken together, these data support the development of anetumab ravtansine for ovarian cancer treatment and highlight its suitability for combination therapy with PLD, carboplatin, copanlisib, or bevacizumab.

## INTRODUCTION

With 239,000 new cases per year, ovarian cancer is the seventh most frequent cancer in women in the world [1]. The highest age-adjusted incidence rates are seen in developed countries. In the United States alone, approximately 14,000 women per year die of ovarian cancer. Most patients are diagnosed at an advanced stage of disease [1]. Patients are initially treated with surgical debulking followed by platinum-based chemotherapy [2–4]. Approximately 75% of patients respond to primary treatment but quickly develop recurrent disease [5–9]. There is a high medical need particularly in recurrent disease and new active treatment modalities beyond chemotherapy are required [2].

Recently, targeted agents have been added as treatment options for ovarian cancer. The vascular endothelial growth factor (VEGF) inhibitor bevacizumab has been approved by the European Medicines Agency (EMA) and the U.S. Food & Drug Administration (FDA) for the treatment of advanced ovarian cancer patients in combination with chemotherapy [10], and the poly (ADP-ribose) polymerase (PARP) inhibitor olaparib has been approved for *BRCA1*/*BRCA2*-mutated high-grade serous ovarian cancer [11, 12].

Mesothelin represents another candidate target for the treatment of ovarian cancer [13–17]. Mesothelin is highly expressed on the surface of tumor cells in various cancers, including ovarian cancer, whereas in normal tissue mesothelin shows limited expression. Mesothelin is frequently co-expressed with and binds to CA125, a well-established ovarian cancer biomarker, and may be involved in the peritoneal spread of ovarian cancer [13, 18, 19]. In epithelial ovarian cancer, high mesothelin expression has been shown to correlate with chemoresistance and poor prognosis [20]. Antibody-based approaches to target mesothelin include the chimeric IgG1 antibody amatuximab, which blocks mesothelin/CA125 interaction [21, 22]. Using ^111^In-labeled amatuximab and single-photon emission computed tomography-computed tomography (SPECT-CT), tumor-specific amatuximab uptake has been demonstrated in mesothelioma and pancreatic cancer patients [23]. Amatuximab was well tolerated in phase 1 clinical studies with stable disease as the best response as monotherapy [24, 25]. The amatuximab-derivative SS1P resulted in pleuritic chest pains as dose-limiting toxicity in phase 1 clinical trials, and only minor tumor responses and stable disease were achieved [26, 27]. Antitumor activity however was limited by neutralizing antibodies [26].

Antibody drug-conjugates (ADCs) consisting of a cytotoxic payload conjugated to an antibody binding to a tumor antigen have demonstrated efficacy in solid tumors [28–30]. The ADC anetumab ravtansine, a fully human anti-mesothelin antibody (MF-T) coupled via a reducible disulfide linker to a microtubule-targeting toxophore DM4, binds to mesothelin with high affinity and delivers the microtubule inhibitor DM4 to mesothelin-positive tumor cells [31]. Anetumab ravtansine has demonstrated potent antitumor activity and good tolerability as single agent in preclinical models including mesothelioma, pancreatic cancer and ovarian cancer [31].

Recent late-stage clinical trials revealed that addition of a third chemotherapy to the standard of care treatment of epithelial ovarian cancer, i.e. carboplatin and paclitaxel, shows increased toxicities without benefit in survival or tumor control [32, 33]. Therefore, targeted agents for the treatment of ovarian cancer either in monotherapy or in combination with chemotherapy should be explored as they may result in increased treatment benefit with more favorable tolerability [34]. Herein, the therapeutic potential of anetumab ravtansine (BAY-94-9343) in ovarian cancer was investigated in monotherapy and in combination with standard of care chemotherapy and targeted agents.

## RESULTS

### Mesothelin is internalized via the endosomal pathway and targeted to lysosomes for degradation

**Figure 1:**
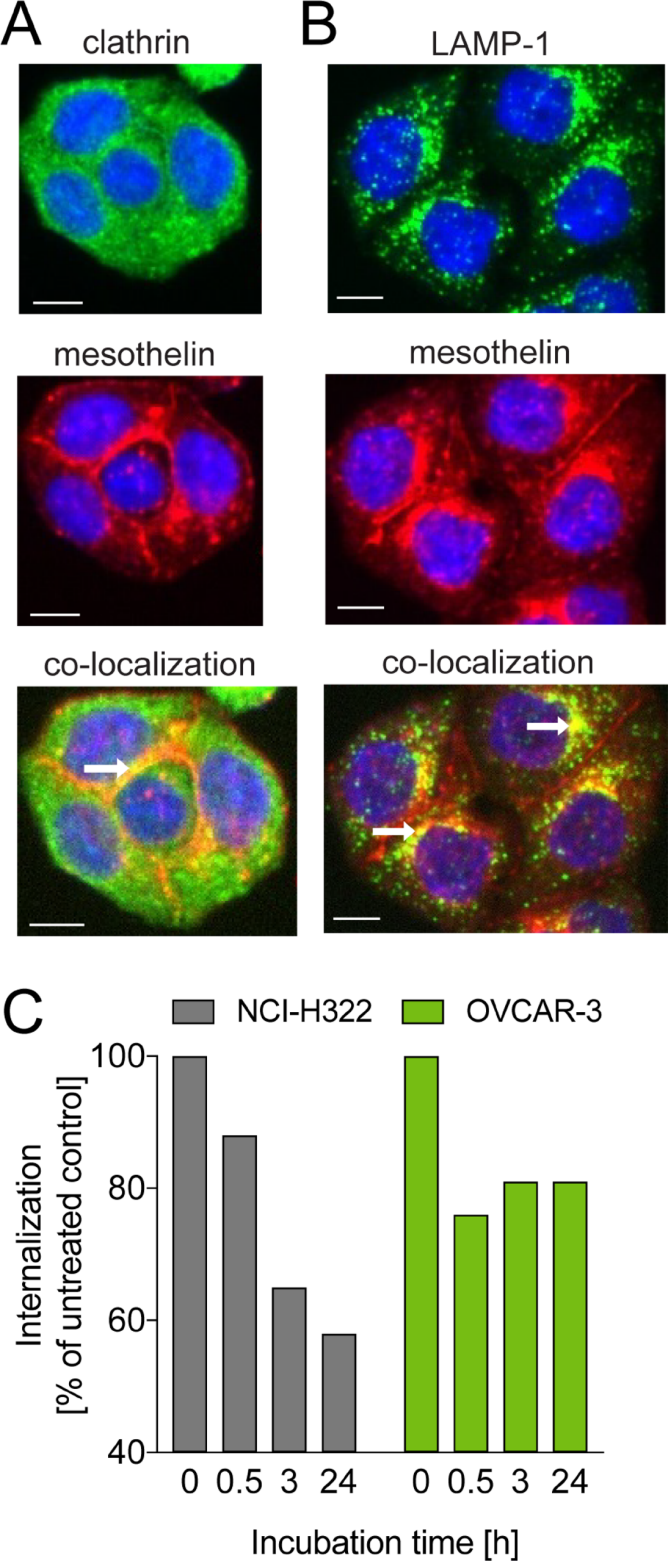
Internalization of the mesothelin-targeted antibody MF-T in cancer cells Co-staining of mesothelin (red) with (**A**) clathrin (green) or (**B**) LAMP-1 (green) in HT29-MSLN colorectal adenocarcinoma cells transfected with human mesothelin as detected by fluorescence microscopy. Co-localization is indicated by yellow fluorescence pointed out by the white arrows. (**C**) Amount of mesothelin on cell surface in NCI-H322 and OVCAR-3 cells treated with the mesothelin-targeted antibody MF-T (anetumab). The data represent a mean of triplicates. All scale bars indicate 10 μm.

To study the internalization of anetumab ravtansine and its localization in cancer cells, the targeting antibody moiety of anetumab ravtansine (anetumab, MF-T) was coupled to a pH-sensitive fluorescent dye and incubated with HT29 human colon adenocarcinoma cells stably transfected with mesothelin (HT29-MSLN). The MF-T co-localized with the endocytosis marker clathrin at the sites of cell-to-cell contacts (Figure 1A). Co-localization with the lysosomal marker lysosome-associated membrane glycoprotein 1 (LAMP-1) was seen in cytoplasmic vesicles (Figure 1B). The MF-T-induced reduction of surface mesothelin expression was confirmed in endogenously mesothelin-positive NCI-H322 human lung cancer and OVCAR-3 human ovarian cancer cells by flow cytometry (Figure 1C).

### Anetumab ravtansine induces mesothelin degradation and re-synthesis of surface mesothelin

**Figure 2:**
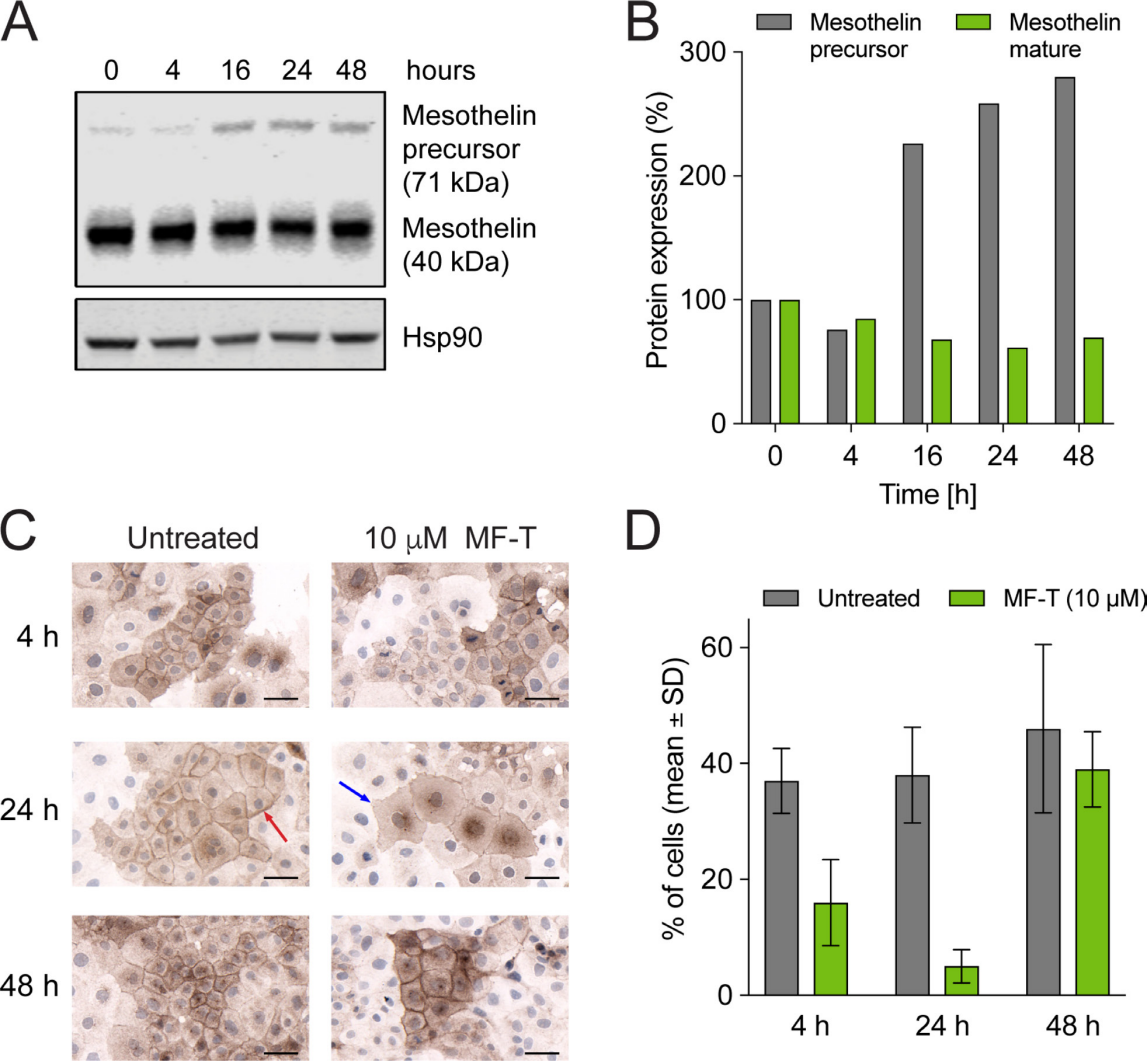
Mesothelin degradation and re-synthesis of surface mesothelin in OVCAR-3 human ovarian cancer cells (**A**) Expression of mature mesothelin and mesothelin precursor was analyzed in OVCAR-3 cells treated with 100 nM anetumab ravtansine using Western blot. HSP90 served as a loading control. (**B**) The percentage of mesothelin expression compared to untreated cells as determined by Western blot described in panel (A). (**C**) Mesothelin expression in OVCAR-3 cells. OVCAR-3 cells were incubated with or without 10 μM anti-mesothelin antibody MF-T and fixed for staining with the anti-mesothelin K1 antibody. (**D**) Mesothelin expression on cell surface upon treatment with anetumab ravtansine. The number of OVCAR-3 cells with a clear membrane signal and the number of all cells per a microscope field were counted. The mean percentage of mesothelin-positive cells in 18 microscope fields per group is shown. All scale bars indicate 50 μm.

To study the mechanism of action of anetumab ravtansine, OVCAR-3 human ovarian cancer cells endogenously expressing mesothelin were incubated with 100 nM anetumab ravtansine for 4, 16, 24 or 48 h and the expression level of mesothelin was detected by Western blot. The OVCAR-3 cells treated with anetumab ravtansine showed decreased mesothelin expression compared to untreated cells, with the highest difference observed at 24 h (Figure 2A, 2B). A higher molecular weight precursor was induced in response to anetumab ravtansine treatment, indicating re-synthesis of mesothelin. The possible degradation and subsequent re-expression of mesothelin on the cell surface was further investigated by incubating OVCAR-3 cells with an excess amount of MF-T for 4, 24 or 48 h (Figure 2D). The cells were stained for mesothelin using the K1 antibody, which recognizes a different epitope than MF-T. At 4 h, strong mesothelin expression was observed on the surface of untreated OVCAR-3 cells (red arrow in Figure 2C). During a 24 h MF-T incubation, mesothelin levels were reduced (Figure 2D) and the cell membrane appeared unstructured (blue arrow in Figure 2C). After 48 h, mesothelin expression on the cell surface increased again, supporting the hypothesis of internalization-induced degradation and subsequent re-synthesis of surface-localized mesothelin by MF-T.

### Anetumab ravtansine induces mitotic arrest, DNA damage and apoptosis *in vitro*

**Figure 3:**
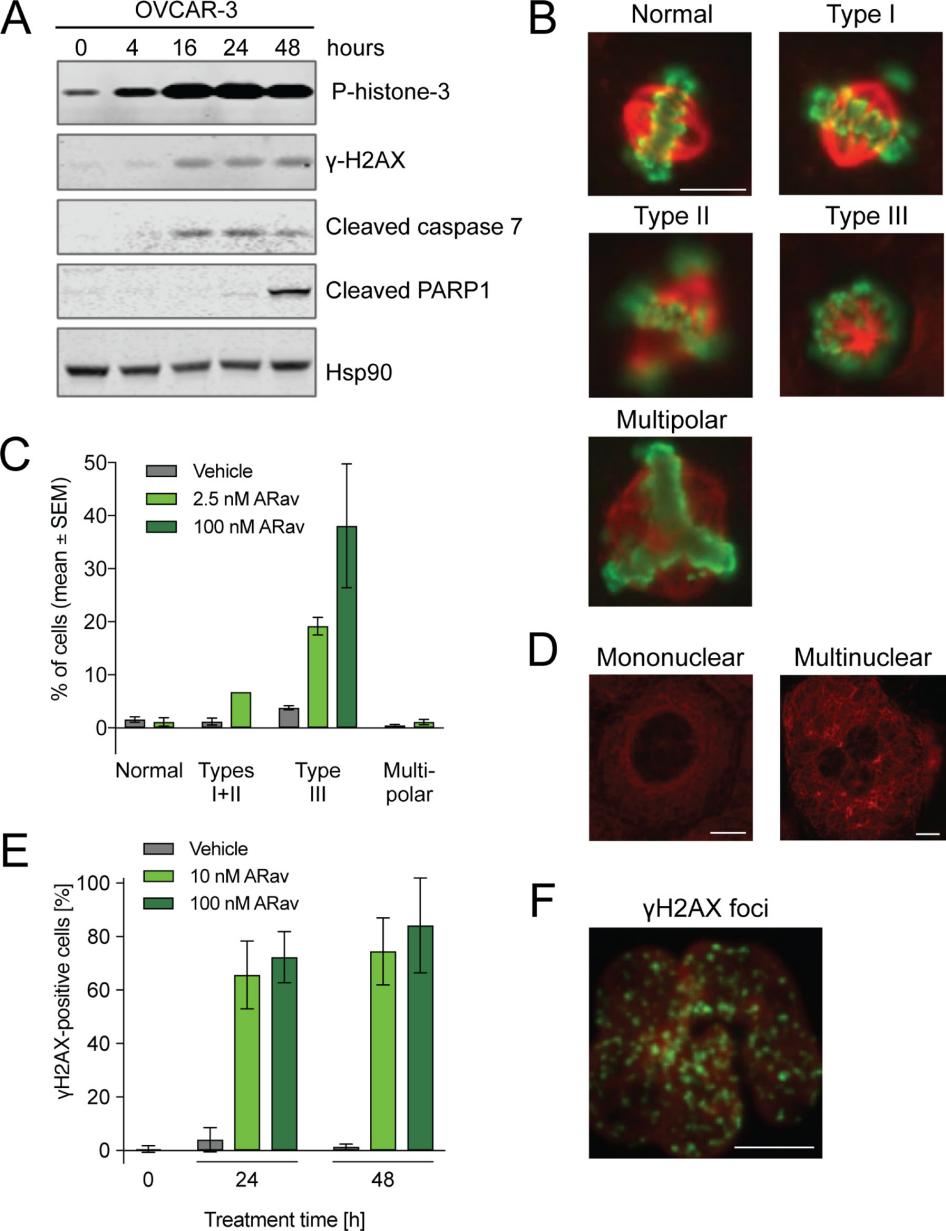
Analysis of anetumab ravtansine mode-of-action in OVCAR-3 human ovarian cancer cells (**A**) OVCAR-3 cells treated with 100 nM anetumab ravtansine were assayed for phospho-histone H3, γH2AX, cleaved caspase 7, cleaved PARP1 and HSP90 by Western blot at indicated time points. (**B**) Representative images from the analysis of mitotic spindle assembly in OVCAR-3 cells treated with 2.5 or 100 nM anetumab ravtansine for 24 h. (**C**) The percentage of cells described in panel B exhibiting mitotic spindles categorized as normal, type I + II (abnormal bipolar mitotic spindles with uncongressed chromosomes), type III (monopolar spindles enclosed in a ball of chromosomes) or multipolar (multipolar spindles) per four microscopy fields. (**D**) Representation of mononuclear and multinuclear OVCAR-3 cells upon treatment with 100 nM anetumab ravtansine for 24 h. (**E**) OVCAR-3 cells were treated with 100 nM anetumab ravtansine for 24 or 48 h and γH2AX was detected by fluorescent microscopy. γH2AX-positive cells as a percentage of all cells (*n* = 6). (**F**) Representative fluorescent microscopy images of γH2AX (green) and DNA (red) staining in cells described in panel E. All scale bars indicate 10 μm. In panel B, the scale bar is representative for all images. ARav, anetumab ravtansine.

The cell damage-inducing capability of anetumab ravtansine was examined in OVCAR-3 cells by detecting markers for mitosis (phospho-histone H3), DNA damage (γH2AX) and apoptosis (caspase 7, PARP1). An increase in the phospho-histone H3 (pHH3) level was detected 16 h after the start of treatment, indicating mitotic arrest of the cells (Figure 3A). Furthermore, anetumab ravtansine treatment induced caspase 7 expression within 16 h after the start of treatment. As expected, the activation of caspase 7 was followed by cleavage of PARP1, one of the downstream targets of caspase 7 [35, 36], 48 h after the onset of anetumab ravtansine treatment. DNA damage was also induced, as indicated by an increased γH2AX signal at 16 h. A minor signal was seen already at 4 h, thus preceding the caspase 7 signal.

Next, the effects of anetumab ravtansine on microtubule (MT) organization and the cell cycle were investigated by fluorescence microscopy. In line with the Western blot results illustrated in Figure 3A, OVCAR-3 cells showed mitotic arrest, indicated by a notable increase in pHH3-positive cells after 24 h of 2.5 nM or 100 nM anetumab ravtansine treatment (Figure 3B). These pHH3-positive cells were frequently separated from each other and showed a round appearance. Of note, the maytansine payload of anetumab ravtansine did not result in the depolymerization of the MT network but rather in alterations of the mitotic spindle organization (Figure 3B–3D). Spindle structures were categorized (normal, type I-III, or multipolar) based on the degree of chromosome alignment [37]. At an anetumab ravtansine concentration of 2.5 nM, which is close to the anti-proliferative IC_50_, the cells showed an increase in chromosome aberration (type I-II) and type III monopolar spindles (Figure 3C). At a higher anetumab ravtansine concentration of 100 nM, almost all cells showed a pHH3-positive type III mitotic phenotype. Following drug exposure, cells in the interphase stage showed more MT bundles and were frequently multinucleated (Figure 3D). Fluorescent microscopy was used to characterize the γH2AX phenotype induced by anetumab ravtansine in OVCAR-3 cells (Figure 3E, 3F). In line with the published results for taxanes [38], we observed an increase of γH2AX foci in the OVCAR-3 cells treated with anetumab ravtansine, suggesting an induction of DNA damage.

### Anetumab ravtansine shows potent *in vitro* efficacy in ovarian cancer cell lines

The antiproliferative activity of anetumab ravtansine was tested in a panel of ovarian cancer cell lines (Table 1). Anetumab ravtansine showed high potency in the tested cell lines, indicated by IC_50_ values in the nanomolar range. In line with *in vitro* data published for other ADCs [39], no linear correlation between the *in vitro* potency and surface mesothelin levels (determined by flow cytometry) could be established (Table 1).

**Table 1:** *In vitro* efficacy of anetumab ravtansine and cell surface mesothelin expression levels in a panel of ovarian cancer cell lines

Cell line	IC_50_ [nM]	Surface mesothelin level (antibodies bound per cell)
A2780	20.7	952
AG6000	15.4	1105
BG1	5.9	900
EFO-21	20.8	9648
IGROV1	41.9	1942
NCI/ADR-RES	42.4	53497
OVCAR-3	10.9	19998
OVCAR-5	11.9	1260
OVCAR-8	32.5	41887
SK-OV-3	4.1	3875

### Potent *in vivo* efficacy of anetumab ravtansine in preclinical mesothelin-positive ovarian cancer models

Next, the *in vivo* antitumor efficacy of anetumab ravtansine was tested in two cell line- and eight patient-derived ovarian cancer models with varying histological backgrounds (Table 2). Anetumab ravtansine was clearly efficacious in five out of ten models tested; in the OVCAR-3 cell line-derived and ST103, ST081, ST207 and ST409 patient-derived xenograft models treatment/control (T/C) ratios between 0 and 0.36 were observed. Anetumab ravtansine resulted in total tumor eradication in ST081 and ST103 PDX models. Furthermore, a response of 100% was observed in ST081, ST103 and ST270 PDX models.

**Table 2:** *In vivo* efficacy of anetumab ravtansine as a monotherapy in a panel of ovarian cancer cell line- and patient-derived xenograft models

Xenograft model	CDX/PDX	Ovarian cancer subtype	T/C (2.5 mg/kg)	IHC score (0–3)	H score (0–300)
ST081	PDX	High-grade serous ovarian cancer	0^a,*^	3	150
ST103	PDX	High-grade serous ovarian cancer	0^a,*^	3	210
ST270	PDX	High-grade serous ovarian cancer	0.04^a,*^	3	185
OVCAR-3	CDX	High-grade serous ovarian cancer	0.17^*^	3	180
ST467	PDX	Serous papillary carcinoma	0.25	2	180
ST409	PDX	High-grade serous ovarian cancer	0.36^*^	3	230
OVCAR-8	CDX	High-grade serous ovarian cancer	0.58^*^	2	175
ST206B	PDX	Serous papillary carcinoma	0.73	2	160
Ov6645	PDX	Malignant mixed Müllerian carcinosarcoma	1.83	0	0
ST2054	PDX	High-grade serous ovarian cancer	1.37	1	50

^a^Anetumab ravtansine administered at 15 mg/kg, Q2W

^*^Significantly different in comparison to vehicle control (*p* < 0.05, *n* = 3–10)

CDX, cell line-derived xenograft; IHC, immunohistochemistry; PDX, patient-derived xenograft; T/C, treatment/control

**Figure 4:**
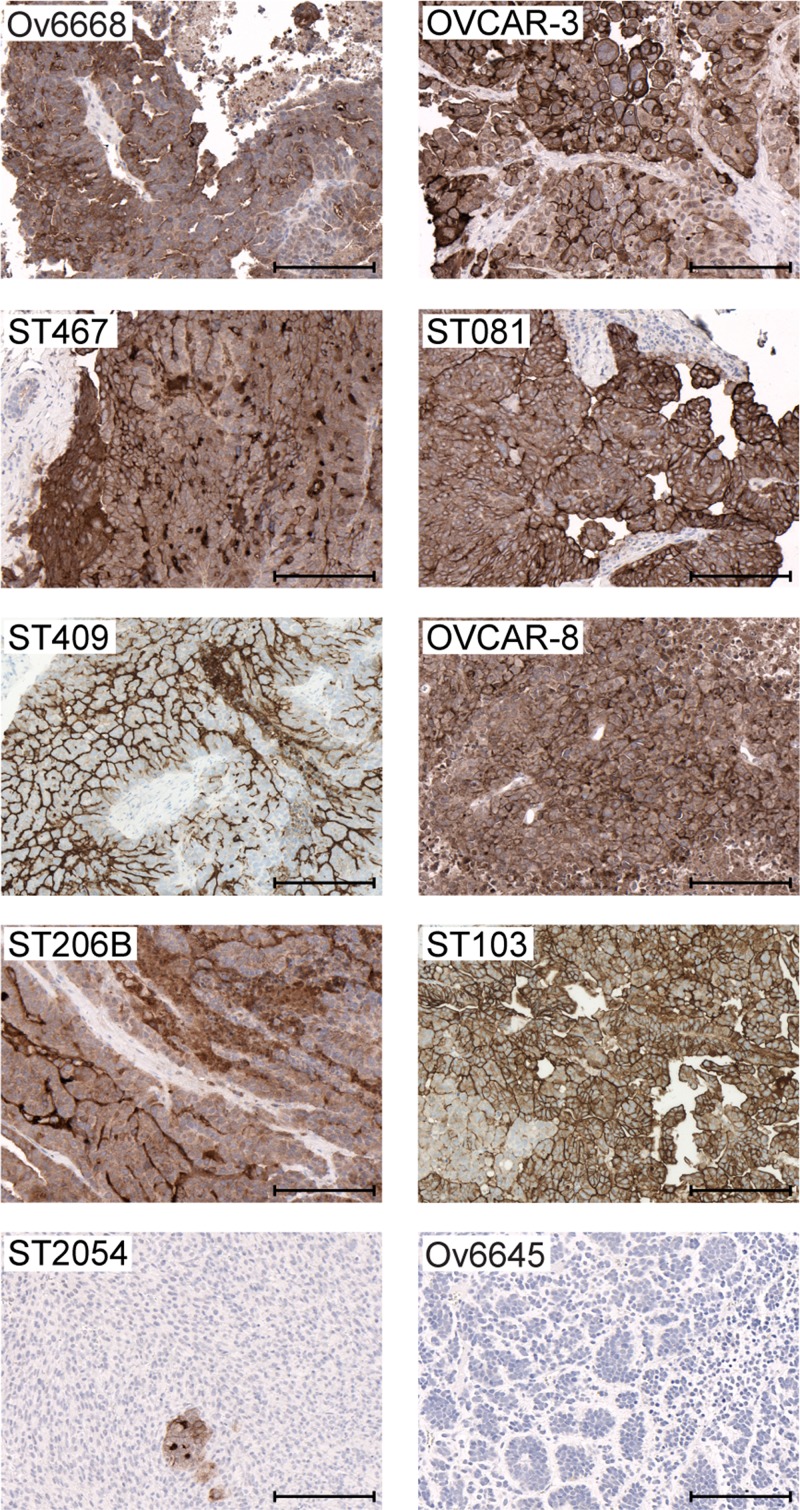
Mesothelin expression in ovarian cancer cell line- and patient-derived xenograft models Representative images of IHC analyses in tumors derived from cell line- (OVCAR-3 and OVCAR-8) and patient-derived (Ov6668, ST467, ST081, ST409, ST206B, ST103, ST054 and Ov6645) xenografts using the anti-MSLN antibody SP74. Brown color indicates mesothelin expression. Scale bars indicate 200 μm.

To investigate the correlation between the antitumor efficacy and mesothelin expression in the ovarian cancer tumor models, histological sections were analyzed by immunohistochemistry (Table 2 and Figure 4). Anetumab ravtansine showed potent anti-tumor activity in models with medium to high mesothelin expression (H-Score >50). In contrast, no efficacy was observed in the mesothelin-negative Ov6645 and ST2054 ovarian cancer PDX models (Table 2 and Figure 4).

### Anetumab ravtansine exhibits improved potency in combination with doxorubicin

**Figure 5:**
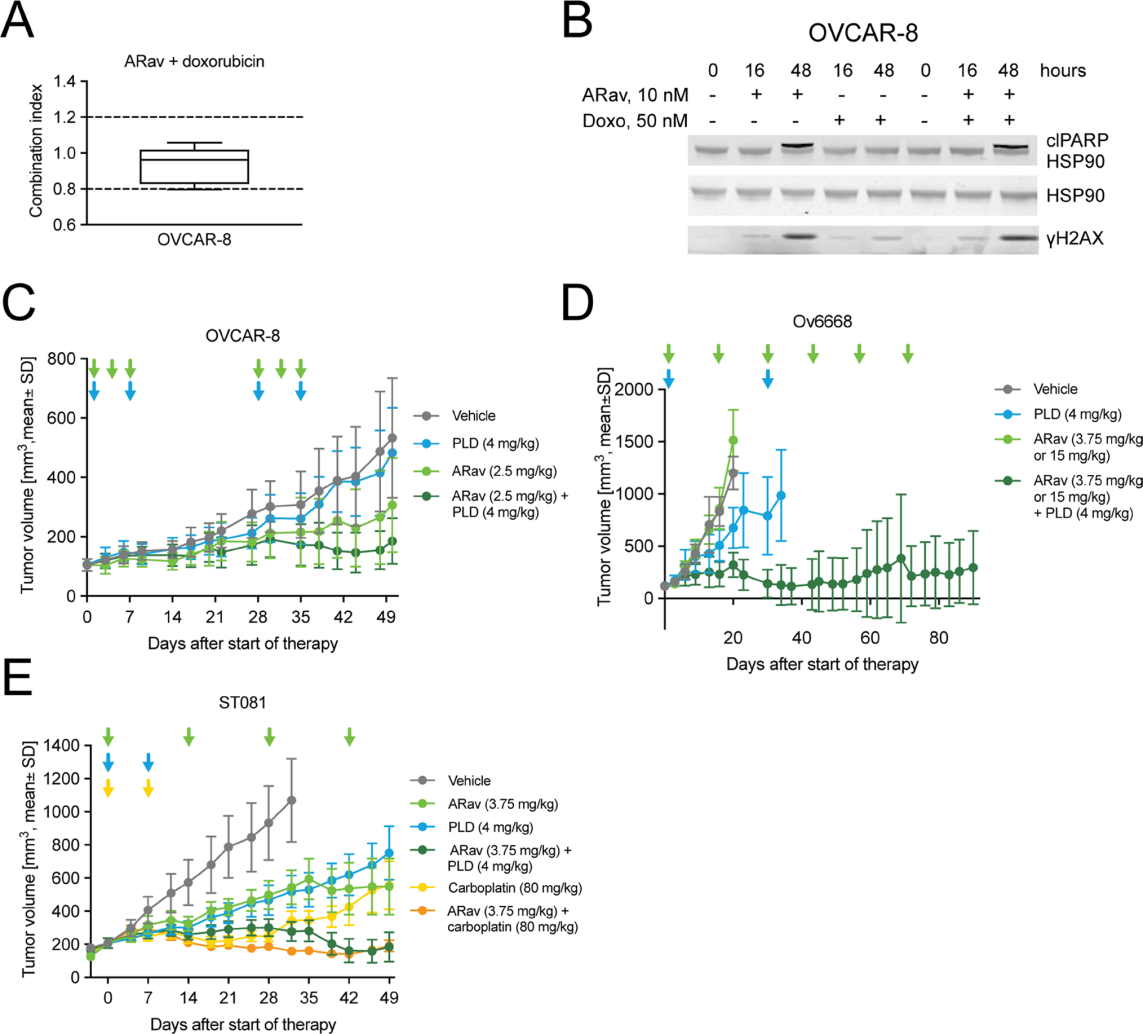
Antitumor efficacy of anetumab ravtansine in combination with doxorubicin/PLD or carboplatin in preclinical ovarian cancer models *in vitro* and *in vivo* (**A**) Combination of anetumab ravtansine with doxorubicin in OVCAR-8 cells *in vitro*. The activity was determined as additive based on the determined combination indices (CI) between 0.8 and 1.2 (*n* = 5). (**B**) OVCAR-8 cell lysates were analyzed for γH2AX, cleaved PARP1 and HSP90 by Western blot at the indicated time points. (**C**) Tumor growth in the OVCAR-8 ovarian cancer model (*n* = 8). Treatments were initiated 21 days after tumor cell inoculation. (**D**) Tumor growth in the Ov6668 ovarian cancer PDX model (*n* = 9). Treatments were initiated 21 days after tumor inoculation. (**E**) Tumor growth in the ST081 ovarian cancer PDX model (*n* = 8). Treatments were initiated when tumors reached a size of 125–250 mm^3^. Anetumab ravtansine (i.v.), PLD (i.v.) and/or carboplatin (i.v.) were administered as indicated by arrows. ARav, anetumab ravtansine; Doxo, doxorubicin.

Pegylated liposomal doxorubicin (PLD) is the clinically most commonly used second-line treatment regimen for recurrent and platinum-resistant ovarian cancer patients. Combination therapies of DNA-damaging agents, such as PLD or carboplatin with microtubule-targeting agents (MTAs) are approved in the treatment of various malignancies including ovarian cancer [40]. Therefore we tested the potential of combining anetumab ravtansine with PLD in ovarian cancer models *in vitro* and *in vivo*. Since doxorubicin is the main active chemical ingredient of PLD and more directly metabolized by tumor cells *in vitro*, it was used in the *in vitro* combination studies. Anetumab ravtansine showed additive interaction with doxorubicin in the ovarian cancer cell line OVCAR-8, characterized by BRCA1 methylation [41], *in vitro*, as indicated by combination indices (CI) between 0.8 and 1.2 [42] in five repeated experiments (Figure 5A). The combination treatment with anetumab ravtansine and doxorubicin resulted in comparable levels of cleaved PARP1 and γH2AX compared to anetumab ravtansine alone (Figure 5B).

The antitumor efficacy of anetumab ravtansine in combination with PLD was further investigated in various ovarian cancer mouse xenograft models, including the OVCAR-8 cell line-derived model, as well as in the Ov6668 and ST081 PDX models. In OVCAR-8 mice, monotherapy with 2.5 mg/kg anetumab ravtansine or 4 mg/kg PLD showed no antitumor activity as indicated by T/C ratios of 0.58 and 0.91, respectively. Combination treatment with anetumab ravtansine and PLD showed improved antitumor activity, resulting in a T/C ratio of 0.35 (*p* < 0.001 vs PLD monotherapy; Figure 5C, Table 3) In the Ov6668 PDX model, the combination of anetumab ravtansine (first dose of 3.75 mg/kg followed by 15 mg/kg, Q2W) with 4 mg/kg PLD was synergistic with improved antitumor efficacy compared to vehicle (T/C = 0.27, *p* < 0.001, day 20) or either one as a monotherapy (anetumab ravtansine, T/C = 1.26; PLD, T/C = 0.56; both *p* < 0.001, day 20; Figure 5D). Five out of nine mice treated with combination therapy (55%) showed complete tumor eradication or tumor shrinkage of more than 30%, whereas no tumor shrinkage was observed in either monotherapy group. The benefit for the combination of anetumab ravtansine with PLD was confirmed in a second PDX model. In the ST081 PDX model, the combination of anetumab ravtansine (3.75 mg/kg) with PLD (4 mg/kg) showed improved antitumor efficacy compared to vehicle (T/C = 0.26, *p* = 0.003) or either of the agents alone (anetumab ravtansine, T/C = 0.51, *p* = 0.030; PLD, T/C = 0.49, *p* = 0.035; Figure 5E).

**Table 3:** *In vivo* efficacy of anetumab ravtansine in combination with other anticancer agents in ovarian cancer cell line- and patient-derived xenograft models

Xenograft model	Treatment^a^	T/C^b^	Statistics^c^	Response^d^	Max. body weight loss (%)^e^
**Combination with PLD**					
OVCAR-8	Vehicle	1.00		10% SD (d50)	0.0
	ARav	0.58	^*^	0% (d50)	0.0
	PLD	0.91	NS	0% (d50)	−0.7
	ARav + PLD	0.35	^***^	20% SD (d50)	0.0
Ov6668	Vehicle	1.00		0% (d40)	0.0
	ARav	1.26	^*^	0% (d40)	0.0
	PLD	0.56	^***^	0% (d57)	0.4
	ARav + PLD	0.27	^***^	11% CR, 44% PR, 11% SD (d110)	0.0
ST081	Vehicle	1.00		0% (d40)	−2.3
	ARav	0.51	NS	12.5% PR (d60)	−1.5
	PLD	0.49	^*^	0% (d60)	−2.0
	ARav + PLD	0.26	^**^	12.5% CR, 50% PR, 12.5% SD (d69)	−4.4
**Combination with copanlisib**					
OVCAR-3	Vehicle	1.00		0% (d71)	−0.1
	ARav	0.17	^***^	11% PR, 33% (d71)	−1.5
	Copanlisib	0.54	^**^	0% (d71)	−5.6
	ARav + copanlisib	0.10	^***^	56% PR, 22% SD (d71)	−5.2
OVCAR-8	Vehicle	1.00		10% SD (d50)	0.0
	ARav	0.59	NS	0% (d50)	0.1
	Copanlisib	0.59	^*^	0% (d50)	−1.3
	ARav + copanlisib	0.46	^***^	0% (d50)	−0.5
**Combination with bevacizumab**					
OVCAR-3	Vehicle	1.00		0% (d71)	−0.1
	ARav	0.17	^***^	11% PR, 33% SD (d71)	−1.5
	Bevacizumab	0.23	^***^	22% SD (d71)	−1.4
	ARav + bevacizumab	0.04	^***^	67% PR (d71)	−1.3
Ov6668	Vehicle	1.00		0% (d40)	0.0
	ARav	1.26	NS	0% (d40)	0.0
	Bevacizumab	0.47	^***^	0% (d63)	0.0
	ARav + bevacizumab	0.36	^***^	89% CR, 11% PR (d110)	0.0
**Combination with carboplatin**					
ST081	Vehicle	1.00		0% (d32)	−2.3
	ARav	0.51	NS	12.5% PR (d60)	−1.5
	Carboplatin	0.32	^**^	12.5% PR (d60)	−2.6
	ARav + carboplatin	0.15	^***^	12.5% PR, 25% SD (d60)	−4.4

^a^Please see Materials and Methods for dosing and schedule.

^b^Treatment/control ratio, calculated from mean tumor volumes on the last day when the vehicle group remained in the study.

^c^Statistical significance in comparison to vehicle control on the last study day of the vehicle group. ^*^p < 0.05; ^**^p < 0.01; ^***^p < 0.001; NS, not significant

^d^Responses calculated based on tumor volumes on the last day of each treatment group.

^e^The maximum mean body weight loss expressed as a percentage of the animal weight at the start of the study. Weight loss greater than 20% is considered toxic.

ARav, anetumab ravtansine; PLD, pegylated liposomal doxorubicin; PR, partial response; SD, stable disease

### Anetumab ravtansine exhibits improved potency in combination with carboplatin

Paclitaxel and carboplatin combination chemotherapy are used as standard of care in first-line therapy of ovarian cancer [43]. Anetumab ravtansine and paclitaxel are both MTAs. We therefore tested if anetumab ravtansine can replace paclitaxel in the treatment of ovarian cancer. The ST081 PDX model was treated with a combination of anetumab ravtansine (3.75 mg/kg) and carboplatin (80 mg/kg). The combination treatment demonstrated improved efficacy compared to vehicle (T/C = 0.15, *p* < 0.0001) or either one as a monotherapy (anetumab ravtansine, T/C = 0.51, *p* = 0.004; carboplatin, T/C = 0.32, *p* < 0.001; Figure 5E).

### Anetumab ravtansine shows enhanced antitumor efficacy when combined with copanlisib

**Figure 6:**
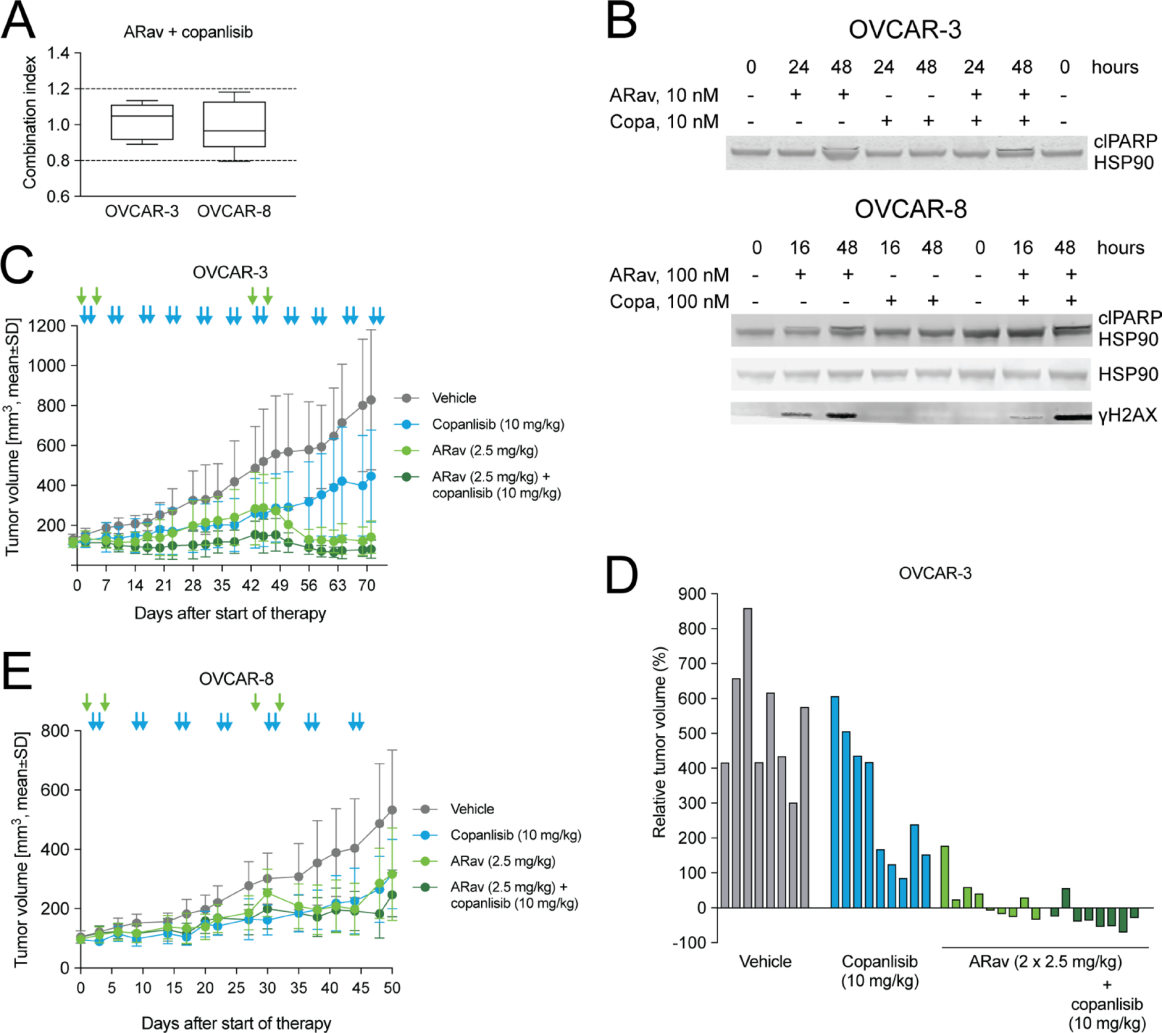
Antitumor efficacy of anetumab ravtansine in combination with copanlisib in preclinical ovarian cancer models *in vitro* and *in vivo* (**A**) Combination of anetumab ravtansine with copanlisib in OVCAR-3 and OVCAR-8 cells *in vitro*. The activity was determined as additive based on the determined combination indices (CI) between 0.8 and 1.2 (*n* = 5). (**B**) Cell lysates were analyzed for γH2AX, cleaved PARP1 and HSP90 by Western blot at the indicated time points. (**C**) Tumor growth in the OVCAR-3 ovarian cancer model (*n* = 9). Treatments were initiated 43 days after tumor cell inoculation. (**D**) Changes in the relative volume of OVCAR-3 tumors in panel A on day 71 after start of therapy, represented as a percentage of the initial tumor volume in each individual mouse. (**E**) Tumor growth in the OVCAR-8 ovarian cancer model (*n* = 7–9). Treatments were initiated 21 days after tumor inoculation. Anetumab ravtansine (i.v.) and/or copanlisib (i.v.) were administered as indicated by arrows. ARav, anetumab ravtansine; Copa, copanlisib.

Copanlisib is a pan-class I phosphoinositide 3-kinase (PI3K) inhibitor with predominant activity on the α and δ isoforms [44, 45] and it was recently approved for the treatment of follicular lymphoma. The PI3K pathway has been identified as one of the most deregulated signaling pathways in many cancers, including ovarian cancer [46]. Anetumab ravtansine showed additive interaction with copanlisib in OVCAR-3 and OVCAR-8 cells *in vitro* (Figure 6A). Furthermore, the combination treatment with anetumab ravtansine and copanlisib resulted in higher effects on DNA damage and apoptosis compared to anetumab ravtansine alone, indicated by increased levels of cleaved PARP1 and γH2AX, respectively (Figure 6B).

Next, the combination of anetumab ravtansine with copanlisib was tested *in vivo* using the OVCAR-3 xenograft model, characterized by an amplification of the AKT2 locus [47]. In this model, monotherapy with 2.5 mg/kg anetumab ravtansine showed efficacy with a T/C ratio of 0.17 (*p* < 0.001) and a stable disease in five out of nine mice (Figure 6C, 6D). The combination of anetumab ravtansine with copanlisib (10 mg/kg) resulted in improved efficacy, as indicated by a partial response in five out of nine mice and improved T/C ratios (Table 3). The combination potential of anetumab ravtansine with copanlisib was tested also in the OVCAR-8 xenograft model. In this study, the combination of 2.5 mg/kg anetumab ravtansine with 10 mg/kg copanlisib was more efficacious than the vehicle (T/C = 0.46, *p* < 0.001); however, no difference in comparison to respective monotherapies was observed (Figure 6E, Table 3).

### Anetumab ravtansine shows improved potency in combination with bevacizumab

**Figure 7:**
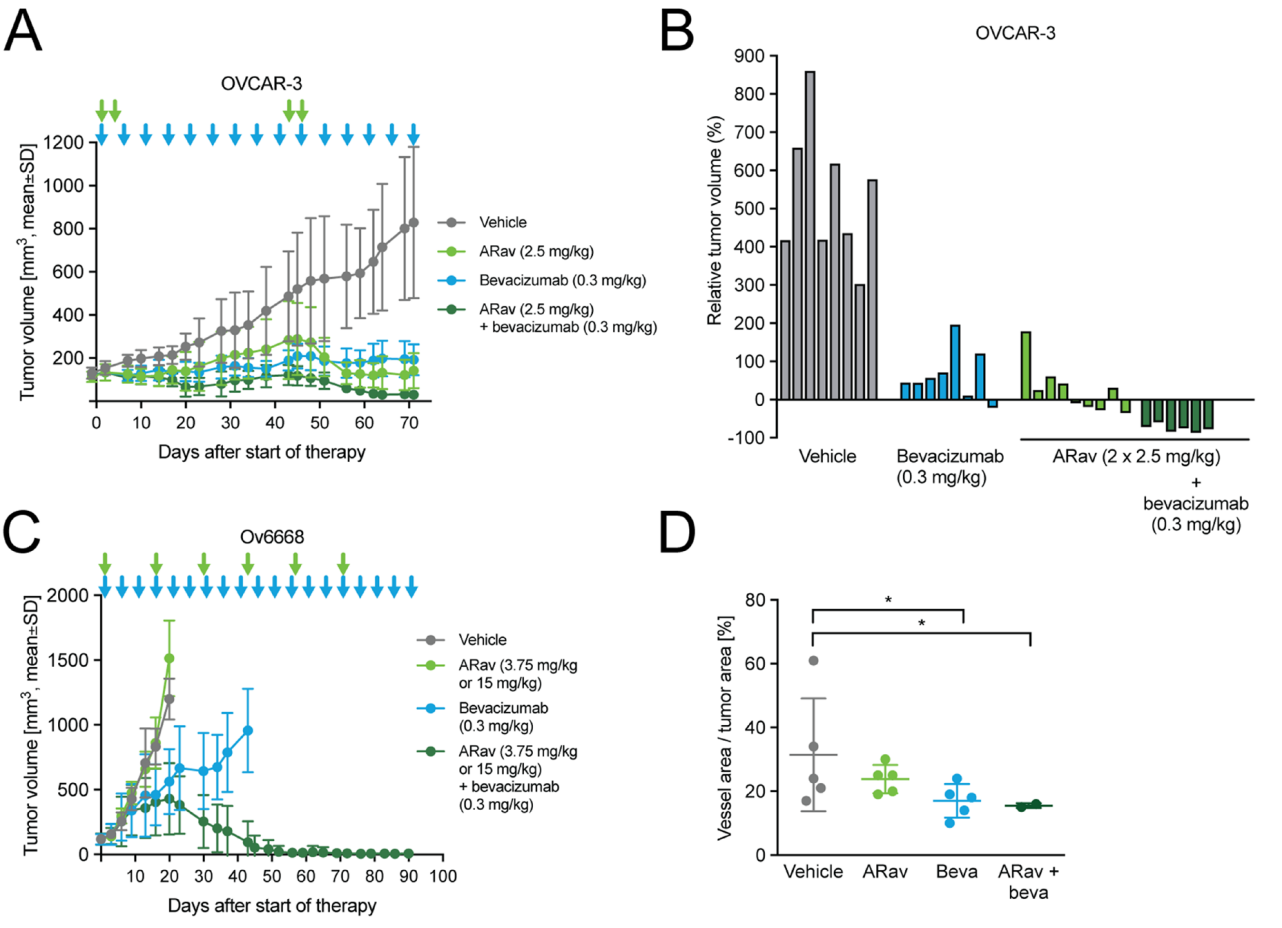
Antitumor efficacy of anetumab ravtansine in combination with bevacizumab in human ovarian cancer xenograft models in mice Anetumab ravtansine (i.v.) and/or bevacizumab (i.p.) were administered as indicated by arrows. (**A**) Tumor growth in the OVCAR-3 ovarian cancer model (*n* = 9). Treatments were initiated 43 days after tumor cell inoculation. (**B**) Changes in the relative volume of OVCAR-3 tumors in panel A on day 71 after start of therapy, represented as a percentage of the initial tumor volume in each individual mouse. (**C**) Tumor growth in the Ov6668 ovarian cancer PDX model (*n* = 9). Treatments were initiated 21 days after tumor inoculation. (**D**) CD31-positive vessel area as a percentage of tumor area in OVCAR-3 mice described in panel A. ARav, anetumab ravtansine; Beva, bevacizumab.

Bevacizumab is the only antiangiogenic therapy approved for the treatment of ovarian cancer patients. The combination of anetumab ravtansine with bevacizumab was tested in the OVCAR-3 and Ov6668 models. In OVCAR-3 mice, monotherapy with anetumab ravtansine or bevacizumab showed significant antitumor efficacy with T/C ratios of 0.17 and 0.23, respectively (both *p* < 0.001; Figure 7A, Table 3). The combination of anetumab ravtansine with bevacizumab was clearly synergistic compared to the respective monotherapies (both *p* = 0.003), resulting in total tumor eradication in eight out of nine mice (Figure 7B). The combination of anetumab ravtansine with bevacizumab was further explored in the Ov6668 PDX model. Bevacizumab (0.3 mg/kg) but not anetumab ravtansine (first dose of 3.75 mg/kg followed by 15 mg/kg) showed significant antitumor activity as monotherapy (T/C = 0.47, *p* < 0.001, day 20; Figure 7C). The combination of anetumab ravtansine with bevacizumab resulted in additive efficacy compared to the respective monotherapies (anetumab ravtansine, T/C = 1.26, *p* < 0.001; bevacizumab, T/C = 0.47, *p* = 0.003). To get a better understanding of the mechanism of action, tumors were harvested at the end of the experiment and stained with the endothelial cell marker CD31. In tumors from vehicle-treated animals numerous large CD31-positive blood vessels were detected. Contrarily, in tumors from bevacizumab-treated animals, fewer CD31-positive blood vessels were detected. Moreover, these vessels were smaller in size, translating into reduced vessel area per tumor area (Figure 7D).

All monotherapy and combination treatments were well-tolerated as indicated by less than 5% body weight losses in the mouse models tested (Table 3).

## DISCUSSION

Cell surface glycoprotein mesothelin is highly expressed in ovarian cancer and its expression has been associated with poor prognosis [48]. In addition, the binding of mesothelin to CA125, an ovarian cancer biomarker, and upregulation of metalloproteinases has been reported to facilitate metastatic spread, making mesothelin an interesting target in ovarian cancer [18, 19, 49, 50]. The ADC anetumab ravtansine takes advantage of the tumor-specific expression of its target antigen mesothelin, thereby delivering the highly cytotoxic microtubule-targeting toxophore DM4 payload to tumor cells [28]. We have previously demonstrated that anetumab ravtansine exhibits superior antitumor efficacy compared to the standard of care cisplatin, resulting in complete tumor eradication in a preclinical ovarian cancer model [31]. Herein, the activity of anetumab ravtansine was investigated in a broader and more diverse set of ovarian cancer models, including patient-derived xenograft models with more relevant disease backgrounds.

Anetumab ravtansine was demonstrated to be rapidly internalized by cancer cells and exhibited the preferred intracellular trafficking route into lysosomes upon binding to mesothelin. Furthermore, anetumab ravtansine showed potent *in vivo* efficacy with T/C ratios less than 0.37 in several cell line- and patient-derived PDX ovarian cancer models. Efficacy was generally higher in tumors with strong mesothelin expression, while no activity was seen in mesothelin-negative ovarian cancer models. These data support the rationale of using mesothelin as a selection marker for patient stratification in clinical studies with anetumab ravtansine.

Despite the availability of platinum-based first-line therapies such as cisplatin and carboplatin for ovarian cancer patients, a high medical need remains for platinum-resistant recurrent ovarian cancer [9]. PLD is the most commonly utilized agent in recurrent ovarian cancer, and it has a response of approximately 26% [51, 52]. Many clinically established cancer therapy regimens combine a DNA-damaging agent with an MTA. MTAs cause prolonged mitotic arrest and partial activation of apoptotic pathways, and thereby DNA damage [38]. Furthermore, MTAs disrupt the intracellular trafficking of DNA repair proteins and thereby can enhance the toxicity of DNA-damaging agents such as PLD/doxorubicin or carboplatin [40]. In line with these reported findings, anetumab ravtansine induced DNA damage in OVCAR-3 and OVCAR-8 cells *in vitro*. The effects were of the same order of magnitude in cells treated with anetumab ravtansine alone or in combination with doxorubicin. Combined treatment with anetumab ravtansine and doxorubicin resulted in additive anti-proliferative activity *in vitro,* presumably through increased DNA damage or complementary effects on microtubule destabilization and DNA damage. The observed *in vitro* efficacy translated into improved *in vivo* efficacy in the anetumab ravtansine and PLD combination therapy in comparison to the respective monotherapies in the OVCAR-8, Ov6668 and ST081 ovarian cancer xenograft models.

A combination of paclitaxel with carboplatin has been the standard of care first-line therapy for ovarian cancer [43]. Based on similar mode-of-actions, there is a rationale to replace paclitaxel with anetumab ravtansine for front-line ovarian cancer therapy. Therefore, we also tested anetumab ravtansine in combination with carboplatin in the ST081 platinum-sensitive ovarian cancer PDX model. Anetumab ravtansine combined with carboplatin resulted in improved efficacy in comparison to the respective monotherapies in the ST081 model. Together, these results provide a rationale for the evaluation of anetumab ravtansine in combination with PLD or carboplatin in ovarian cancer patients.

Activation of the PI3K/AKT signaling pathway can mediate resistance to cytotoxic agents in ovarian cancer [53]. Preclinical data demonstrate the improved activity of paclitaxel, an MTA used as the first-line therapy in ovarian cancer [2], in combination with inhibitors of the PI3K/AKT pathway [53, 54]. In clinical studies, however, the combination of PI3K inhibitors and paclitaxel has been limited by the toxicity of these agents [55, 56]. ADCs are associated with less side effects and an improved risk-benefit ratio compared to paclitaxel, and therefore, present an alternative, more promising combination partner for PI3K inhibitors. Herein, the combination of anetumab ravtansine with copanlisib, a pan-class I PI3K inhibitor, showed improved antitumor activity compared to the respective monotherapies and was well-tolerated in the OVCAR-3 model, characterized by an amplification of the AKT2 locus [47]. In OVCAR-3 cells, combination treatment with anetumab ravtansine and copanlisib resulted in the induction of the apoptosis markers PARP and γH2AX *in vitro*, supporting PI3K-mediated inhibition of apoptosis as a potential mechanism of action.

Bevacizumab is a targeted anticancer therapy and currently the only antibody-based therapy approved for use in ovarian cancer [57]. Bevacizumab was approved based on improved progression-free survival; however, no advantage in overall survival has been demonstrated [58]. Combining bevacizumab with other targeted agents has been presented as an attractive approach to improve therapeutic efficacy [58], e.g. by improving the tumor penetration of antibody-based therapies by normalizing the aberrant structure and function of tumor vasculature [59]. In the present study, the combination of anetumab ravtansine with bevacizumab resulted in improved antitumor efficacy in the OVCAR-3 model. It is noteworthy that tumor shrinkage was seen in the combination group only. The superior activity of the combination treatment was confirmed in the Ov6668 ovarian cancer PDX model, supporting further exploration of the combination of these two targeted agents.

Overall, this work supports the development of anetumab ravtansine as monotherapy or in combination with various targeted agents and chemotherapy in the treatment of ovarian cancer. A phase 1b study (NCT02751918) exploring the pharmacokinetics and maximum tolerated dose of anetumab ravtansine in combination with PLD in ovarian cancer patients is currently ongoing.

## MATERIALS AND METHODS

### Cells and compounds

The ovarian cancer cell lines were obtained from ATCC, NCI, ECACC or Public Health England and authenticated by the DSMZ using short tandem repeat (STR) DNA fingerprinting. NCI-H322 human lung cancer cells were obtained from ATCC. Cells were passaged for up to 6 months after receipt or resuscitation and maintained at 37° C in a humidified atmosphere containing 5 % CO_2_. OVCAR-3 and OVCAR-8 cells were cultured in RPMI-1640 medium (Biochrom) supplemented with 10% (w/v) fetal calf serum (FCS). All other cell lines were cultured in DMEM/F12, GlutaMAX (Invitrogen) supplemented with 10% (v/v) heat-inactivated FCS (Sigma) and antibiotics (Invitrogen). Anetumab ravtansine (BAY 94–9343), the anti-mesothelin antibody MF-T (BAY 86–1903) and the transfected HT29-MSLN cell line stably expressing mesothelin were produced as described previously [31].

### Internalization

To investigate the internalization mechanism of anetumab ravtansine, the targeting antibody moiety (MF-T, BAY 86–1903) of the ADC was coupled to a pH-sensitive fluorescent dye. Mesothelin-expressing HT29-MSLN cells were incubated for 6 h with the anti-mesothelin antibody MF-T or with an isotype control. Cells were fixed and permeabilized with methanol, followed by staining with antibodies against either the endocytosis marker clathrin or the lysosomal marker LAMP-1 (lysosome-associated membrane glycoprotein 1) and analyzed by fluorescence microscopy using the InCellAnalyzer 1000 (GE Healthcare).

Cell surface mesothelin expression was evaluated in NCI-H322 human lung cancer and OVCAR-3 human ovarian cancer cells endogenously expressing mesothelin. Cells were incubated with the anti-mesothelin antibody MF-T (10 µg/ml) for 0.5, 3 or 24 h and analyzed for mesothelin expression by flow cytometry (FACS).

To study the mechanism of action of anetumab ravtansine, OVCAR-3 human ovarian cancer cells endogenously expressing mesothelin were incubated with 100 nM anetumab ravtansine for 4, 16, 24 or 48 h and the expression level of mesothelin was detected by Western blot (please see details below).

### Mitotic progression

To evaluate cell cycle stage and microtubule organization, 24 × 10^3^ OVCAR-3 cells/well were seeded in chamber slides and incubated for 24 h. Cells were treated with anetumab ravtansine for 24 h, fixed in 4% paraformaldehyde for 20 min at 4° C and washed twice with cold PBS. Cells were stained with the PathScan Apoptosis and Proliferation Multiplex IF kit (Cell Signaling Technology) following the manufacturer’s instructions. The primary antibody was incubated overnight at 4° C. Stained cells were mounted using Fluorescent Mounting Medium (Dako) and imaged with an Axio Scan-Z1 (Zeiss) fluorescence microscope.

### Proliferation assay

The analysis of anetumab ravtansine efficacy in a panel of ovarian cancer cell lines was performed at Charles River Discovery Research Services Germany GmbH. Ovarian cancer cells were seeded on 96-well plates, and after 24 h, the cells were treated with anetumab ravtansine (5 mg/ml, corresponding to 33.3 µM) or DMSO in 10 different concentrations with half-log increments up to 1000 pM. The cells were incubated for 72 h, and cell viability was determined using the CellTiter-Blue^®^ Cell Viability Assay (Promega). The data was analyzed by a 4-parameter nonlinear curve fit (Oncotest Data Warehouse software).

### *In vitro* combination assays

OVCAR-3 and OVCAR-8 cells (300 to 500 cells/well) were seeded in 384-well plates. Anetumab ravtansine (300 nM–0.41 nM) and copanlisib or doxorubicin (both 3 µM–0.41 nM) were in mixed compound ratios of 0.2, 0.4, 0.5, 0.6, 0.8 and 1. Cells were incubated for 72 h and viability was measured using Cell Titer-Glo^®^ (Promega) according to the manufacturer’s instructions. IC_50_ values were calculated for each individual combination data point, and the respective isobolograms were generated. Combination indices (CI) were calculated according to the median-effect model [60]. Activity was determined as synergistic with CI < 0.8, additive with 0.8 < CI > 1.2 and antagonistic with CI > 1.2.

### Detection of mesothelin expression

Mesothelin expression was determined immunohistochemically (IHC) in tumors fixed in neutral buffered formalin and embedded in paraffin. Following epitope retrieval at pH 9, freshly cut 3 µm slides were stained with 0.5 µg/mL anti-MSLN antibody SP74 (Spring Bioscience). Membrane mesothelin staining was scored by a trained pathologist. The percentage of tumor cells staining positive for mesothelin at each intensity level (0, 1, 2, or 3) and an H-score were calculated as described previously [61]. The K1 anti-mesothelin antibody (Thermo Fisher) was used for immunocytochemistry in OVCAR-3 cells.

Cell surface mesothelin expression levels were determined in a panel of ovarian cancer cell lines by FACS. Cells were seeded in 96-well round-bottom microtiter plates at a density of 3.6 × 10^5^ cells/well and incubated with MF-T (0.1, 0.25, 0.5, 1, 3, 5 µg) or isotype control (5 µg) antibodies for 2 h at 4° C in the dark. After the incubation, the cells were washed with FACS staining buffer and resuspended for analysis. Instrument settings were set up with QuantiBRITE beads (BD Biosciences) according to the manufacturer´s instructions. Flow cytometric analysis was carried out on the Attune NxT Acoustic Focusing Cytometer and analyzed using the FlowJo software.

In the *in vitro* studies, protein expression was determined in OVCAR-3 and OVCAR-8 cells by Western blot. Cells were incubated with 10 or 100 nM anetumab ravtansine or copanlisib, or with 50 or 500 nM doxorubicin. In parallel, cells were treated with combinations of anetumab ravtansine (10 and 100 nM) and copanlisib (10 and 100 nM), or anetumab ravtansine (10 and 100 nM) and doxorubicin (50 and 500 nM). Culture medium was used as vehicle control. At 0,16, 24 and 48 h, cells were harvested for protein isolation. The cell extracts (10 µg of total protein) were separated by sodium dodecyl sulfate polyacrylamide gel electrophoresis (SDS-PAGE) and transferred to nitrocellulose membranes. The membranes were blocked in 5% skim milk (in TBS-Tween, 0.1 % (v/v)) and incubated with the indicated primary antibodies (mesothelin (D9R5G), phospho-histone H3 (Ser10), phospho-Akt (Ser473), cleaved PARP and phospho-p44/42MAPK (Erk1/2); all rabbit origin (Cell Signaling); HSP90 (mouse origin, BD Bioscience). Proteins were detected using IRDye-labeled secondary antibodies with reactivity against rabbit and mouse (IRDye 800CW anti-rabbit and IRDye 680LT; Licor) and visualized using the Odyssey infrared imaging system (Licor).

### *In vivo* tumor models

All animal experiments were conducted in accordance with the German animal welfare law and approved by local authorities. For the OVCAR-3 and OVCAR-8 xenograft models, tumor cells (8 × 10^6^ or 2 × 10^6^, respectively) in Matrigel^®^ (Basement Membrane Matrix, BD Biosciences) were inoculated subcutaneously to the right lower flank region of female nude/nude mice (Janvier Labs). The *in vivo* studies with the ovarian cancer patient-derived xenograft (PDX) models were performed at EPO Berlin-Buch GmbH, Germany (Ov6645 and Ov6668) or START, San Antonio, TX, USA (ST270, ST467, ST081, ST409, ST206B and ST2054). Ovarian cancer tissue pieces (2 × 2 mm), obtained from *in vivo* passage, were implanted subcutaneously in the inguinal region of female nude/nude or Scid (for the ST103 model) mice. Tumor volume [(length × width^2^)/2] and body weight were measured by caliper at least twice weekly, and the treatment response was defined using the RECIST criteria [62]. Progressive disease (PD) was defined as greater than 20% increase in tumor size. Partial response (PR) was defined as greater than 30% reduction in tumor size. Complete response (CR) was defined as an absence of any palpable tumor mass. No tumor growth or a slight reduction (<30%) or small increase (<20%) in tumor size was defined as stable disease (SD). Treatment-to-control (T/C) ratios were calculated on the basis of mean tumor volume. In the monotherapy experiments shown in Table 2, anetumab ravtansine was administered intravenously (i.v.) at 2.5 mg/kg three times every third day (Q3Dx3). For the *in vivo* combination studies with pegylated liposomal doxorubicin (PLD, Doxil^®^) or copanlisib (Bayer AG) in OVCAR-8 xenografts, anetumab ravtansine was administered i.v. at 2.5 mg/kg on days 1, 4, 7, 28, 32 and 35, or on days 1, 4, 28 and 35, respectively. For the *in vivo* combination studies in OVCAR-3 xenografts, anetumab ravtansine was administered i.v. at 2.5 mg/kg on days 1, 4, 43 and 46. For the Ov6668 xenografts, anetumab ravtansine was administered i.v. at 3.75 mg/kg on day 1 and at 15 mg/kg on days 16, 30, 43, 57 and 71. For the ST081 xenografts, anetumab ravtansine was administered i.v. at 3.75 mg/kg every second week (Q2W). PLD was administered i.v. at 4 mg/kg on days 1, 7, 28 and 35 (OVCAR-8 xenografts), on days 1 and 30 (Ov6668 xenografts) or on days 0 and 7 (ST081 xenografts). Carboplatin (Paraplatin^®^) was administered i.v. at 80 mg/kg QWx2. Copanlisib was administered at 10 mg/kg, 2 days on/5 days off, i.v., starting on day 2. Bevacizumab (Avastin^®^) was administered intraperitoneally (i.p.) at 0.3 mg/kg, every fifth day (Q5D).

### Statistical analyses

All analyses were performed using the statistical programming language R (version 3.5.0) and results were considered significant at *p*-value < 0.05. Validity of the model assumptions was checked for each fitted statistical model. Analyses were performed using linear models estimated with generalized least squares that included separate variance parameters for each study group. For *in vivo* monotherapy studies, mean comparisons between the treatment and the control group were performed using the estimated linear model. For *in vivo* combination studies, all datasets were analyzed using second order linear mixed effects models with random intercepts and slopes for each subject. Pairwise contrasts were corrected for familywise false positive rate using Tukey’s, Sidak’s or Dunnet’s method where appropriate. All longitudinal models included the first and second order fixed time effects for each group and random intercepts and slopes for each subject.

## Abbreviations

ADC: antibody drug-conjugate
ARav: anetumab ravtansine
ATCC: American Type Culture Collection
CA125: cancer antigen 125
CI: combination index
DSMZ: Deutsche Sammlung von Mikroorganismen und Zellkulturen GmbH
ECACC: European Collection of Authenticated Cell Cultures
EMA: European Medicines Agency
FCS: fetal calf serum
FACS: fluorescence-activated cell sorting
FDA: US Food & Drug Administration
IHC: immunohistochemistry
LAMP-1: lysosome-associated membrane glycoprotein 1
MF-T: mesothelin antibody
MSLN: mesothelin
MT: microtubule
MTA: microtubule-targeting agent
NCI: National Cancer Institute
PARP1: poly(ADP-ribose) polymerase 1
pHH3: phospho-histone H3
PDX: patient-derived xenograft
PI3K: phosphoinositide 3-kinase
PLD: pegylated liposomal doxorubicin
RPMI: Roswell Park Memorial Institute
SDS-PAGE: sodium dodecyl sulfate polyacrylamide gel electrophoresis
SPECT-CT: single-photon emission computed tomography-computed tomography
STR: short tandem repeat
T/C: treatment/control
VEGF: vascular endothelial growth factor
Q3D: every third day
Q5D: every fifth day
Q2W: every second week
QW: once weekly
